# Ondansetron causing near fatal catastrophe in a renal transplant recipient

**DOI:** 10.4103/0019-5049.76582

**Published:** 2011

**Authors:** Sandeep Sahu, Sunaina Tejpal Karna, Anil Agarwal, Sushil Prakash Ambesh, Aneesh Srivastava

**Affiliations:** Department of Anaesthesiology, Sanjay Gandhi Post Graduate Institute of Medical Sciences, Lucknow, Uttar Pradesh, India; 1 Department of Urology and Renal Transplant, Sanjay Gandhi Post Graduate Institute of Medical Sciences, Lucknow, Uttar Pradesh, India

Sir,

Ondansetron, a selective 5-HT3 antagonist used effectively for the prevention and treatment of post operative nausea and vomiting,, has been reported to cause adverse cardiovascular events.[1] We are reporting a very rare adverse effect of severe bradycardia and hypotension after giving ondansetron 4 mg intravenously.

A female patient aged 27 years, with chronic kidney disease, was scheduled for pre-emptive renal transplant. She was hypertensive and was controlled on oral clonidine, prazosin and nifidipine. Preoperative examinations and investigation were within normal limits, except haemoglobin of 8 g%, urea 56 mg/dl, creatinine 4.5 mg/dl and mild pulmonary arterial hypertension. In the operation theatre, the patient was connected to monitoring devices. Left radial artery cannulation was secured for invasive arterial pressure (IABP) monitoring and right internal jugular vein for central venous pressure monitoring under local anaesthesia. Balanced general anaesthesia technique used for induction and maintence as per protocol. Following induction, the patient received injection methylprednisolone (500 mg) for intra-operative immuno-suppression. The induction of anaesthesia and the intra-operative period were uneventful. After kidney transplantation on releasing the clamp, the patient had good diuresis. Before closure of the laparotomy incision, ondansetron intravenous 4 mg was administered as antiemetic prophylaxis. Within 3 min of its administration, patient’s heart rate dropped from 84 to 30/min and IABP decreased from 140/90 to 32/20 mmHg. Electrocardiogram showed sinus bradycardia with QT interval prolongation (17 mm). Inspired oxygen concentration (FiO_2_) was increased to 1% and chest compressions were started at a rate of 100/min. Immediately atropine 0.6 mg was administered intravenously. Within 2-3 min, the heart rate returned to 100/min with IABP 150/90 mmHg. Serial arterial blood gas (ABG) during the event showed a fall in pH from 7.32 to 7.17 with a base deficit of –10. Nothing active was done and pH gradually increased to 7.34 spontaneously.

After the completion of the surgery, with stable haemodynamic and normal ABG, the patient was extubated on the table as per protocol. She was fully awake, oriented with no neurological deficit and with stable haemodynamics. She was monitored in the kidney transplant unit. After 18 days of the uneventful stay, she was discharged with the advice for follow-up in nephrology.

On reviewing the literature, it was found that the submicromolecular affinity of ondansetron like that of droperidol for K+ channels encoded by “human ether-a go-go related gene” (HERG) is possibly responsible for the prolongation of cardiac repolarisation, thus resulting in conduction disturbances like QT/QTc interval prolongation and their theoretical proarrhythmic potential.[2] Animal studies have demonstrated that 5HT receptors present on endings of vagal afferent nerves, especially in the left ventricle, are implicated in causing bradycardia via the von Bezold Jarisch reflex. Being a 5-HT3 antagonist, ondansetron inhibits this reflex causing tachycardia. Even though tachyarrhythmias and atrial fibrillation have been described in the literature in past, reports of bradyarrythmias are rare.[3,4] The cardiovascular effects of serotonin receptors are complex and consist of bradycardia or tachycardia, hypotension or hypertension, and vasoconstriction or vasodilatation. Thus, in any patient, the blockade of 5-HT3 receptors by ondansetron will produce effects dependent on the pre-existing serotonergic activity in both arms of the autonomic nervous system.[5]

The presence of chronic kidney disease, hypertension and multiple antihypertensive medications influences the pre-existing serotonergic activity in the transplant recipient. All these can alter the response to 5-HT3 antagonists. A prolonged QT interval is a risk factor for atrial and ventricular arrhythmias and sudden death.[6] Individuals with occult/congenital QT prolongation are at risk of experiencing malignant dysrhythmias when ondansetron is administered; especially in conjunction with anaesthetic agents like opioids and inhalational anaesthetics, muscle relaxants like atracurium associated with or without atropine significantly prolong the QT interval.[7] The renal transplant recipients are pathophysiologicaly very complex cases, and there are many possible aetiologies for cardiac compromise in these patients. these includes compression of the inferior vena cava, infusion of ischaemic metabolites from the transfused kidney, air embolus even a simple syringe swap and remote possibility of peritoneal stimulation resulting in vagal bradycardia even at the end of a procedure, is a relatively potential causes of such type event.

In conclusion, we recommend judicious administration of ondansetron with availability of emergency resuscitation drugs and equipment along with meticulous monitoring.
